# Cushing Disease Mimicking Ectopic Adrenocorticotropic Hormone–Secreting Disease

**DOI:** 10.1016/j.aed.2025.06.001

**Published:** 2025-06-10

**Authors:** David Louis Fisher, Merav Fraenkel, Lior Baraf

**Affiliations:** 1 Endocrinology Department, Soroka University Medical Center, Beer Sheva, Israel; 2 The Faculty of Health Sciences, Ben-Gurion University of the Negev, Beer Sheva, Israel; 3 Department of Endocrinology and Metabolism, Clalit Community Health Services, Jerusalem District, Israel

**Keywords:** Cushing syndrome, Cushing disease, ectopic ACTH-secreting tumor

## Abstract

**Background/Objective:**

We present the case of a patient whose initial biochemical results suggested an ectopic adrenocorticotropic hormone (ACTH)–secreting tumor, with markedly increased serum ACTH levels, increased 24-hour urinary free cortisol levels, and hypokalemia. Although these features are more commonly associated with ectopic ACTH secretion, the patient was ultimately diagnosed with Cushing disease

**Case Report:**

A 46-year-old man presented with recurrent headaches and progressive skin darkening. He had hypertension and a body mass index of 28.4 kg/m^2^. Initial blood work revealed hypokalemia at 3.2 mEq/L (normal reference range, 3.5-5.1 mEq/L), and screening tests for Cushing syndrome were pathologic including a highly increased urinary free cortisol level (605 μg/24 h; normal reference range, 20.9-292 μg/24 h). The serum ACTH level was high (89 pg/mL; normal reference range, 0-45.4 pg/mL), and pituitary magnetic resonance imaging showed an 8-mm lesion in the pituitary gland. Transsphenoidal pituitary surgery was performed with subsequent postsurgical remission.

**Discussion:**

Higher urinary and plasma cortisol levels are typical of ectopic ACTH-secreting tumors and likely cause hypokalemia and hypertension, which are encountered more frequently in patients with ectopic ACTH-secreting tumors. In the absence of a pituitary mass 1 cm or more in size, further testing to differentiate Cushing disease from an ectopic source is required.

**Conclusion:**

Highly increased serum ACTH and cortisol levels, hypokalemia, and hypertension occur more frequently in patients with ectopic ACTH-secreting tumors. However, we show that they may occur in Cushing disease, and therefore, it would be wrong to assume a diagnosis of an ectopic ACTH-secreting tumor based on these findings alone.

**Highlights**

• Hypertension and hypokalemia are associated with ectopic adrenocorticotropic hormone–secreting tumors
• Hypertension and hypokalemia occur less frequently in Cushing disease
• When investigating hypercortisolemia, a diagnostic pathway should be followed

**Clinical Relevance**

This case demonstrates the importance of following a diagnostic pathway when investigating hypercortisolemia. Highly increased serum adrenocorticotropic hormone (ACTH) and urinary cortisol levels, hypertension, and hypokalemia are typically associated with ectopic ACTH-secreting tumors. We show that they can occur in Cushing disease, and therefore, an ectopic ACTH-secreting tumor should not be assumed.

## Introduction

Endogenous Cushing syndrome has an incidence of 1.8 to 3.2 per million^1^^,^^2^ and may be divided into adrenocorticotropic hormone (ACTH)–dependent (70%-80%) and ACTH-independent (20%-30%) diseases. ACTH-dependent disease may be further divided into Cushing disease and ectopic ACTH-secreting tumors (60%-70% and 5%-10% of endogenous Cushing syndrome cases, respectively).^3^

Hypertension is found in 80% of adult patients with Cushing syndrome;^4^ however, it is rare for patients with hypertension to be diagnosed with Cushing syndrome, occurring in just 0.5% to 1% of patients with hypertension.^5^^,^^6^ Hypokalemia and hypertension occur more frequently in patients with ectopic ACTH-secreting tumors than in patients with Cushing disease.^7^, ^8^, ^9^, ^10^, ^11^

We present the case of a 46-year-old man who ultimately was diagnosed with Cushing disease. However, the initial biochemical results implied a diagnosis of an ectopic ACTH-secreting tumor, which demonstrated the variable presentation of Cushing syndrome and the importance of following a diagnostic pathway when investigating the source of endogenous hypercortisolism.

## Case Report

A 46-year-old man with a previous diagnosis of obstructive sleep apnea treated with continuous positive airway pressure presented with recurrent headache and progressive darkening of his skin. These symptoms were accompanied by fatigue and bilateral shin swelling that had been present for the previous few months.

Two months earlier, he had been diagnosed with hypertension; treatment with ramipril was started, and the dose escalated to 7.5 mg/day. He did not take any other medications. On physical examination, he had a blood pressure of 163/93 mm Hg and a heart rate of 97 bpm. His weight was 88.5 kg, and he had a body mass index of 28.4 kg/m^2^.

He had a generalized tan hue to his skin (although it was winter) and bilateral pedal pitting edema, which extended up to the middle of his shins. He was noted to have leg alopecia. No other features of Cushing syndrome were noticed including proximal myopathy, facial plethora, or evidence of bruising or striae. The rest of his cardiovascular, respiratory, and abdominal examinations were unremarkable.

Over the course of the following 2 months, the dose of ramipril was increased to 10 mg/d, and treatment with amlodipine was added (first 5 mg/d, which was subsequently escalated to 10 mg/d). To minimize the number of tablets that the patient needed to take, amlodipine and ramipril were both stopped, and a single daily combination pill containing amlodipine 10 mg and valsartan 160 mg was given. Three months after his original presentation, his blood presentation remained elevated; therefore, a third-line agent, doxazosin 1 mg/d, was added, which was then increased to 1 mg twice a day. This combination of treatments resulted in normotension.

Initial blood test results are shown in the Table. Testing revealed hypernatremia (147.0 mEq/L; normal reference range, 135-145 mEq/L), hypokalemia (3.2 mEq/L; normal reference range, 3.5-5.1 mEq/L), and an increased Lactate dehydrogenase level (863.0 U/L; normal reference range, 240-480 U/L).

**Table:** Initial Blood Test Results

Test	Result	Reference range
White blood count (K/μL)	6.6	4.5-11
Hemoglobin (g/dL)	13.4	13.5-17.5
Hematocrit (%)	40.4	41-53
Platelet (K/μL)	211	150-450
Lymphocytes (K/μL)	0.9	1-4.8
Neutrophils (K/μL)	4.9	1.8-7.7
Glucose (mg/dL)	86	70-100
Urea (mg/dL)	38	17-43
Creatinine (mg/dL)	0.9	0.7-12
Sodium (mEq/L)	147	135-145
Potassium (mEq/L)	3.2	3.5-5.1
Alkaline phosphatase (U/L)	79	30-120
Aspartate aminotransferase (U/L)	34	0-35
Alanine transaminase (U/L)	52	0-45
Gamma-glutamyl transferase (U/L)	45	0-55
Total bilirubin (mg/dL)	0.6	0.3-1.2
Lactate dehydrogenase (U/L)	863	240-480
Transferrin (mg/dL)	221	200-360
Ferritin (ng/mL)	334	20-250
Total cholesterol (mg/dL)	249	<200
Triglycerides (mg/dL)	97	<150
High-density lipoprotein (mg/dL)	61	>40
Low-density lipoprotein (mg/dL)	169	<100

Serum cortisol following an overnight 1-mg dexamethasone suppression test (ODST) measured 38.0 μg/dL (normal reference range, <1.8 μg/dL), and a 24-hour urinary free cortisol (UFC) collection measured 605 μg/24 h (normal reference range, 20.9-292 μg/24 h). ACTH measured 113 pg/mL (normal reference range, 0-45.4 pg/mL).

Screening for other pituitary endocrinopathies revealed a low thyroxine level of 0.83 ng/dL (normal reference range, 0.89-1.76 ng/dL) and inappropriately normal levels of thyroid-stimulating hormone, which suggested central hypothyroidism. The triiodothyronine level was 0.2 ng/dL (normal reference range, 0.2-4.2 ng/dL). Screening also revealed hypogonadotropic hypogonadism with a suppressed luteinizing hormone level at 0.9 IU/L (normal reference range, 1.5-9.3 IU/L) and low total testosterone level at 1.2 ng/mL (normal reference range, 2.7-9.2 ng/mL). The follicle-stimulating hormone level was 3.8 IU/L (normal reference range for men, 1.4-18.1), the insulin-like growth factor level was 154 ng/mL (normal reference range, 67.3-205.8 ng/mL), and the prolactin level was 179 mU/L (normal reference range, 45-375 mU/L). The serum renin level was 20.1 μIU/mL (normal reference range, 5.3-99.1 μIU/mL), and the aldosterone level was below the reference range.

Pituitary magnetic resonance imaging (MRI) showed a 7.5 × 8–mm well-defined lesion in the left lateral aspect of the pituitary gland compatible with an adenoma (Fig.). The mass abutted the medial aspect of the left internal carotid artery but did not impinge on the optic chiasm.

**Fig:**
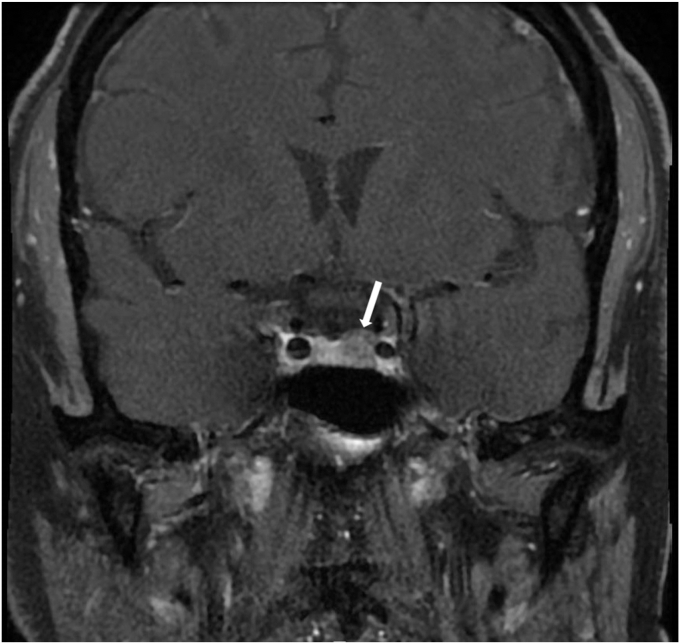
T1 magnetic resonance coronal view of the pituitary sella showing a mass (indicated by arrow) in the left lateral aspect of the pituitary gland.

A high-dose 8-mg dexamethasone suppression test was performed, which resulted in a fall of greater than 50% in the serum cortisol level, from 24.9 μg/dL to 6.45 μg/dL.

Urinary metanephrines and serum calcitonin levels were measured and were normal. A computed tomography (CT) scan of the chest showed no evidence of a lung tumor, and a CT scan of the abdomen and pelvis did not find any evidence of a neuroendocrine tumor.

A working diagnosis of pituitary Cushing disease was made, and the patient was referred for transsphenoidal pituitary surgery, which was performed without any complications.

The cortisol levels measured 12.3 μg/dL (normal reference range, 3-25 μg/dL) on the first postoperative day and 4.0 μg/dL (normal reference range, 3-25 μg/dL) on the second postoperative day. Glucocorticoid replacement therapy was started with prednisone 5 mg/d.

On histopathologic analysis, a gray, brittle tissue was noted. Microscopically, an epithelial tumor composed of basophilic cells was seen with typical architecture suggestive of a neuroendocrine pituitary tumor. The tumor cells were immunohistochemically stained. There was strong staining for cytokeratin 8/18 and weak staining for T-box transcription factor, somatostatin receptor 2A, ACTH, chromogranin, synaptophysin, and periodic acid-Schiff (PAS). The marker MIB1 was seen in 3% of the cells. It was concluded that despite the weak staining for ACTH and considering the basophilic appearance and the strong staining for PAS and cytokeratin filaments, the results support a diagnosis of a densely granulated corticotroph pituitary neuroendocrine tumor.

After surgery, the patient felt malaise. There were no symptoms of headache, polyuria, or polydipsia. His weight had increased to 92 kg before surgery and then decreased to 85 kg 6 months after surgery. His pedal edema gradually abated, and his hyperpigmentation resolved. The antihypertensive medications were gradually stopped, and he remained normotensive. Blood biochemistry testing showed normalization of potassium levels. The central hypothyroidism and hypogonadotropic hypogonadism seen before surgery all resolved. Seven weeks after being discharged from hospital with prednisolone 5 mg, the steroid regime was changed to hydrocortisone 10 mg twice a day, which was then weaned and subsequently stopped 8 months postoperatively. Three years after surgery, he remains well without any signs or symptoms of Cushing disease.

## Discussion

We describe a case of Cushing disease presenting with hypertension and hypokalemia that resolved after transsphenoidal resection of the pituitary adenoma. Excised cells stained strongly for PAS and cytokeratin 8/18, which strongly supported a corticotroph cell origin, although Crooke hyaline changes, which is thought to occur in up to 83% of Cushing disease cases, were not seen.^12^ A reduction in the postoperative serum cortisol level to 4 μg/dL on the second postoperative day was predictive of long-term disease remission.^13^

An ACTH level greater than 84.5 pg/mL supports a diagnosis of ectopic ACTH secretion with a sensitivity of 83% and specificity of 75%.^8^ In addition, the preoperative serum cortisol level is often higher in patients with ectopic ACTH-secreting tumors compared with patients with Cushing disease,^11^ and it has been suggested that a UFC result greater than 471 μg/24 h (normal reference range, 20.9-292 μg/24 h) strongly indicates an ectopic source.^14^ Urinary and serum cortisol levels inversely correlate with serum potassium,^15^ and hypokalemia, which can occur in patients with Cushing disease, occurs more frequently in patients with ectopic ACTH-secreting tumors.^8^, ^9^, ^10^, ^11^ Hypertension is also more prevalent in patients with ectopic ACTH-secreting tumors than in those with ACTH-independent Cushing syndrome from an adrenal source and those with Cushing disease.^7^ Normally, 11-beta-hydroxysteroid dehydrogenase type 2 enzyme, located in the renal tubule, converts bioactive cortisol into inactive cortisone.^16^ Excessively secreted cortisol in Cushing syndrome may saturate the 11-beta -hydroxysteroid dehydrogenase type 2 enzyme and bind to the mineralocorticoid receptor, leading to increased urinary potassium excretion in exchange for sodium, which leads to hypokalemia, metabolic acidosis, and hypertension.^8^ This suggestion was supported by an in vitro study that showed that the conversion of cortisol to cortisone was inhibited when cells were incubated with corticosterone^.16^ Patients with ectopic ACTH-secreting tumors usually have higher levels of serum cortisol, which may bind to the mineralocorticoid receptor resulting in a higher prevalence of hypokalemia and hypertension than in patients with Cushing disease.^11^^,^^17^

Patients with clinical presentations those are suspicious for Cushing syndrome should be screened biochemically. Testing consists of a combination of a UFC test, a late-night salivary cortisol test, or an ODST. With the exception of ODST, tests should be repeated for confirmation.^18^ After diagnosis, a blood test for ACTH is the next step. If the ACTH level is normal or high, a pituitary MRI is performed to search for an adenoma. In the absence of an adenoma or in the presence of a small adenoma less than 6 mm in size, further testing to differentiate Cushing disease from an ectopic ACTH-secreting tumor is performed, consisting of a corticotropin-releasing hormone and/or desmopressin stimulation test along with a whole-body CT, or the “gold standard” test, invasive inferior petrosal sinus sampling (IPSS).^18^ In the most recent guideline update, there was no consensus regarding the workup of lesions 6 to 9 mm in size, although the majority supported performing IPSS.^18^

In our patient, despite having Cushing disease, his ACTH level was 133 pg/mL, and his 24-hour UFC level was 605 μg/24 h (normal reference range, 20.9-292 μg/24 h), and he presented with hypertension, which required 3 classes of antihypertensive medications, and hypokalemia, all of which pointed toward the presence of an ectopic ACTH-secreting tumor, although Cushing disease could not be excluded. A diagnostic pathway was initially followed including performing a head MRI, which revealed an 8-mm mass. Because the mass was less than 1 cm in size, Cushing disease could not be assumed, and an ectopic ACTH-secreting tumor continued to be suspected. However, serum cortisol was suppressed by high-dose dexamethasone by more than 50%, a test that has a sensitivity of 80.8% to diagnose Cushing disease.^19^ In addition, tests for pheochromocytoma and medullary thyroid cancer were normal, and CT scanning of the chest, abdomen, and pelvis found no evidence of a neuroendocrine tumor, which made a diagnosis of ectopic ACTH-secreting tumor unlikely. Therefore, it was opted not to perform invasive IPSS and instead to proceed to pituitary surgery, a decision that was not out of line with the consensus guideline where there is no consensus regarding the optimal diagnostic workup for a lesion of this size.^18^

This case demonstrates the variable presentation of Cushing syndrome and the importance of following a diagnostic pathway when investigating the source of endogenous cortisol excess to facilitate a timely diagnosis and to avoid a delay in definitive management. Hypertension and hypokalemia are typically associated with ectopic ACTH-secreting tumors. However, we show that they can occur in patients with Cushing disease, and therefore, the presence of an ectopic ACTH-secreting tumor should not be assumed.

## Disclosure

The authors have no conflicts of interest to disclose.
